# Diagnostic performances and unnecessary US-FNA rates of various TIRADS after application of equal size thresholds

**DOI:** 10.1038/s41598-020-67543-z

**Published:** 2020-06-30

**Authors:** Sun Huh, Hye Sun Lee, Jiyoung Yoon, Eun-Kyung Kim, Hee Jung Moon, Jung Hyun Yoon, Vivian Youngjean Park, Jin Young Kwak

**Affiliations:** 1 Department of Radiology, Research Institute of Radiological Science, and Center for Clinical Imaging Data Science, Yonsei University College of Medicine, Seoul, Korea; 2 Biostatistics Collaboration Unit, Yonsei University College of Medicine, Seoul, Korea

**Keywords:** Head and neck cancer, Diagnosis

## Abstract

We compared the diagnostic performances and unnecessary FNA rates of several guidelines and modified versions using the size threshold of the ACR TIRADS. Our Institutional Review Board approved this retrospective study and waived the requirement for informed consent and all methods were performed in accordance with the Declaration of Helsinki. A total of 1,384 thyroid nodules in 1,301 patients with definitive cytopathologic findings were included. US categories were assigned according to each guideline. We applied the size threshold suggested by the ACR TIRADS for FNA to the Kwak, ATA and EU guidelines and defined these modified guidelines as the modified Kwak (mKwak), modified ATA (mATA) and modified EU (mEU) guidelines. Diagnostic performances and unnecessary FNA rates of all guidelines were evaluated. Of 1,384 thyroid nodules, 291 (21%) were malignant. Among the original guidelines, the ACR TIRADS had the highest specificity, accuracy, LR and AUC (62.2%, 66%, 2.128 and 0.713). The mKwak, mATA and mEU guidelines had higher specificity, accuracy, LR and AUC (P < 0.001 for all), and fewer unnecessary FNAs, compared with their original guidelines. Among all original and modified guidelines, the mKwak guideline had the highest specificity, accuracy, LR and AUC (64%, 68.6%, 2.389 and 0.75). The unnecessary FNA rate was the lowest with the mKwak guideline (61.1%). The highest sensitivity was observed with the ATA guideline (98.6%). After incorporating the size threshold of the ACR TIRADS to other TIRADS, all guidelines showed higher diagnostic accuracy and lower unnecessary FNA rates than their original versions. The mKwak guideline showed the best diagnostic performances.

## Introduction

Thyroid ultrasonography (US) is now regularly performed in clinical practice and thyroid nodules are exceedingly common on US with as many as 68% of adults having one, leading to issues of overdiagnosis and overtreatment^1,2^. Many guidelines recommend fine-needle aspiration (FNA) based on several risk stratification systems which use different US features and even different size thresholds^3–7^. Current risk stratification systems using US features can be broadly divided into two types: the point-scale Thyroid Imaging Reporting and Data System (TIRADS) suggested by Kwak et al. ^8^, Park et al. ^9^ and the American College of Radiology (ACR)^3^ and the pattern-recognition TIRADS suggested by Horvath et al. ^10^, the 2015 American Thyroid Association (ATA)^7^, and European Thyroid Association (EU)^11^. Different size criteria have been suggested by the ATA guideline, ACR and EU TIRADS^3,7,11^. Although there are many guidelines for recommending FNA for thyroid nodules on US, a worldwide communicable system does not presently exist.

Recently, Grani et al. ^12^ demonstrated that the ACR TIRADS reduced unnecessary FNAs more than other international guidelines with a very low false-negative rate (2.2%, 6/268). The ACR TIRADS suggests a higher size threshold for FNA than other guidelines while still recommending similar malignancy risks for each final assessment category^3,7,11^, and this higher size threshold is thought to explain the decrease in unnecessary FNAs^3^. However, physicians may need more time to classify a nodule on US when using the ACR TIRADS because each US feature is weighted differently^3^. On the other hand, one of other point-scale risk stratification systems proposed by Kwak et al. (Kwak TIRADS) has been proven to be practical and easily applicable in the assessment of thyroid nodules^8,13–20^, and can be performed by simply counting the number of suspicious US features without considering the malignancy probability of each US feature. One recent study compared the diagnostic efficiency of Kwak and ACR TIRADS and found the former to have higher AUC and accuracy^19^. However, the study did not consider the size threshold for recommending FNA^19^. We assumed that if they have similar diagnostic performances with the same size threshold for thyroid nodules, radiologists and clinicians can choose the more convenient risk stratification system for daily practice.

To find an effective guideline for recommending FNA for thyroid nodules, we investigated the diagnostic performances and unnecessary FNA rates of several guidelines in their original form, and their modified versions using the size threshold proposed by the ACR TIRADS.

## Results

### Baseline clinicopathological characteristics

**Figure 1:**
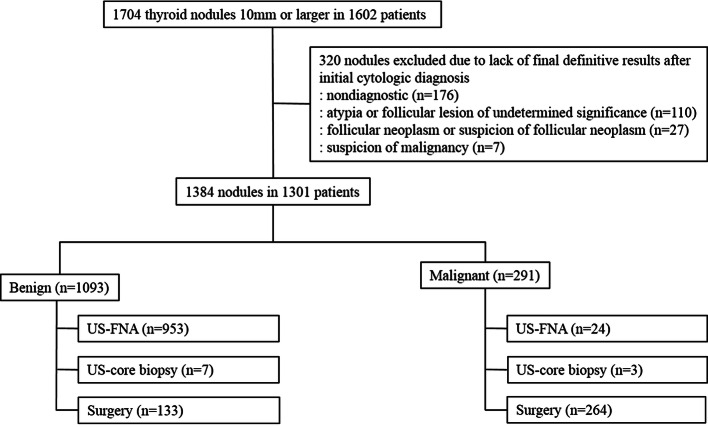
Diagram of the study cohort. *FNA* fine-needle aspiration, *US* ultrasonography.

Of 1,384 thyroid nodules, 1,093 (79%) were benign and 291 (21%) were malignant (Fig. 1, Table 1). 397 nodules (28.7%) underwent surgery, 10 nodules (0.7%) were diagnosed by core needle biopsy and the last 977 (70.6%) nodules were diagnosed by cytologic findings from FNA. Among the 397 nodules which underwent surgery, 264 (66.5%, 264/397) were diagnosed as malignant and 133 (33.5%, 133/397) as benign. The malignant nodules were comprised of 234 papillary thyroid carcinomas (197 conventional, 33 follicular, 2 solid, 1 columnar and 1 oncocytic variant), 21 minimally invasive follicular carcinomas, 5 medullary carcinomas, 3 anaplastic carcinomas and 1 metastatic nasopharyngeal carcinoma. The most frequently excised benign nodules were follicular adenoma (n = 70) followed by adenomatous hyperplasia (n = 59), Hurthle cell adenoma (n = 3), and fibrotic nodule (n = 1). Demographics and US features of the patients and nodules are summarized in Table 1. The mean age (mean 51.1 ± 13.4; range, 18–90) was significantly higher in patients with benign nodules than patients with malignant nodules (mean 47 ± 13.7 years; range, 18–85 years) (P < 0.001). Malignant thyroid nodules were significantly smaller than benign nodules (mean diameter 20.3 ± 12.9 mm and 24 ± 12.3 mm, respectively) (P < 0.001). The malignant thyroid nodules had significantly higher rates of solid composition, hypoechogenicity or marked hypoechogenicity, microlobulated or irregular margins, microcalcifications or mixed calcifications, and nonparallel shape than benign nodules (P < 0.001 for all).

**Table 1:** Demographics of patients and nodules.

	**Final pathology**		**Total**	**Malignancy rate**	**P value**
	**Benign (n = 1,093)**	**Malignant (n = 291)**			
No. of nodules	1,093 (79)	291 (21)	1,384		
No. of patients	1,024 (78.7)	277 (21.3)	1,301		
Age					< 0.001
Mean ± SD	51.1 ± 13.4	47 ± 13.7	50.2 ± 13.6		
Range	18–90	18–85	18–90		
Sex					0.111
Men	179 (17.5)	60 (21.7)	239 (18.4)		
Women	845 (82.5)	217 (78.4)	1,062 (81.6)		
Size					< 0.001
Mean ± SD	24 ± 12.3	20.3 ± 12.9	23.2 ± 12.6		
Range	10–100	10–70	10–100		
US feature					
Composition					< 0.001
Solid	554 (50.7)	252 (86.6)	806 (58.2)	31.27 (1.7)	
Predominantly solid	417 (38.2)	35 (12)	452 (32.7)	7.74 (1.3)	
Predominantly cyst	122 (11.2)	4 (1.4)	126 (9.1)	3.18 (1.6)	
Echogenicity					< 0.001
Marked hypoechoic	18 (1.7)	36 (12.4)	54 (3.9)	66.67 (6.6)	
Hypoechoic	370 (33.9)	206 (70.8)	576 (41.6)	35.76 (2.1)	
Iso- to hyperechoic	705 (64.5)	49 (16.8)	754 (54.5)	6.5 (1)	
Margin					< 0.001
Well	939 (85.9)	83 (28.5)	1,022 (73.8)	8.12 (0.9)	
Microlobulated or irregular	154 (14.1)	208 (71.5)	362 (26.2)	57.46 (2.7)	
Calcification					< 0.001
Negative	909 (83.2)	119 (40.9)	1,028 (74.3)	11.58 (1.1)	
Macro or eggshell	135 (12.4)	35 (12)	170 (12.3)	20.59 (3.2)	
Micro or mixed	49 (4.5)	137 (47.1)	186 (13.4)	73.66 (3.3)	
Shape					< 0.001
Parallel	1,044 (95.5)	178 (61.2)	1,222 (88.3)	14.57 (1.1)	
Nonparallel	49 (4.5)	113 (38.8)	162 (11.7)	69.75 (3.7)	

Data in parentheses are percentages.

*SD* standard deviation.

### Malignancy rates according to categories in the risk stratification systems

Each risk stratification system had significantly different malignancy rates according to categories (Table 2, P < 0.001 for all). Most of the categorized lesions according to ACR and EU TIRADS were all in the range of the recommended risks of malignancy except for the not suspicious lesions (category 2) of ACR TIRADS and low risk (category 3) lesions of EU TIRADS. All categories except nodules of intermediate suspicion (category 4) in the ATA guideline were outside the recommended range.

**Table 2:** Comparison of Malignancy Rates with Several Risk Stratification Systems.

	**Category**	**Final diagnosis**		**P value**	**Calculated risk of malignancy (%)**	**Recommended risk of malignancy (%)**
		**Benign (n = 1,093)**	**Malignant (n = 291)**			
ACR	2—not suspicious	355 (32.5)	15 (5.2)	< 0.001	4.1	2
	3—mildly suspicious	337 (30.8)	16 (5.5)		4.5	5
	4—moderately suspicious	318 (29.1)	66 (22.7)		17.2	5–20
	5—highly suspicious	83 (7.6)	194 (66.7)		70	≥ 20
Kwak	3—no suspicious US feature	382 (35)	15 (5.2)	< 0.001	3.8	
	4a—one suspicious US feature	387 (35.4)	21 (7.2)		5.2	
	4b—two suspicious US features	201 (18.4)	41 (14.1)		16.9	
	4c—three or four suspicious US features	116 (10.6)	160 (55)		58	
	5—five suspicious US features	7 (0.6)	54 (18.6)	< 0.001	89	
ATA	2—very low suspicion	485 (44.4)	20 (6.9)		4	< 3
	3—low suspicion	260 (23.8)	13 (4.5)		4.8	5–10
	4—intermediate suspicion	215 (19.7)	52 (17.9)		19.5	10–20
	5—high suspicion	133 (12.2)	206 (70.8)		60.8	> 70–90
EU	3—low risk	642 (58.7)	28 (9.6)	< 0.001	4.2	2–4
	4—intermediate risk	247 (22.6)	33 (11.3)		11.8	6–17
	5—high risk	204 (18.7)	230 (79)		53	26–87

Data in parentheses are percentages.

*ACR* American College of Radiology^3^, *Kwak* Kwak et al.’s study^8^, *ATA* American Thyroid Association^7^, *EU* European Thyroid Association^11^.

### Diagnostic performances of the guidelines

**Figure 2:**
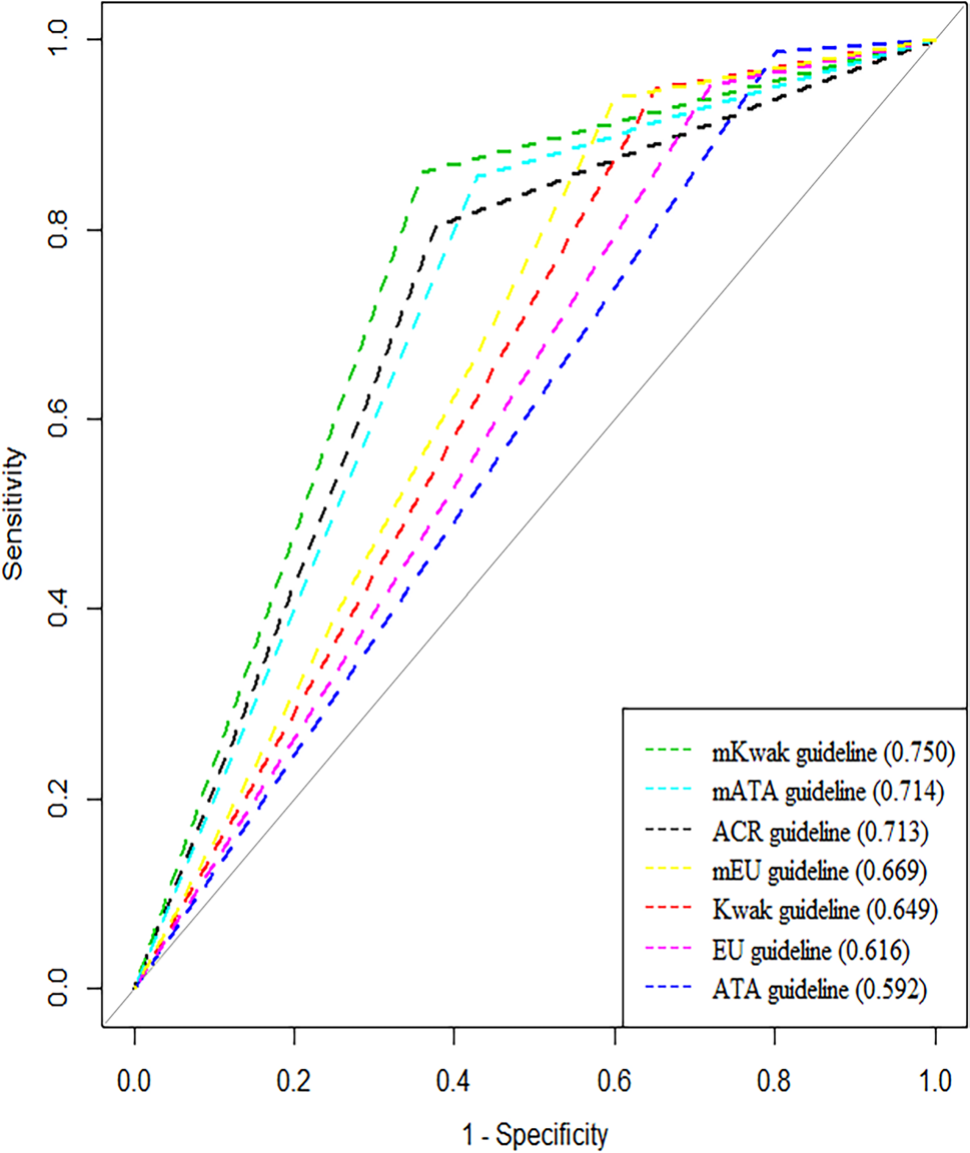
Receiver operating characteristic curves of the four guidelines and their modified guidelines. The modified Kwak (mKwak), modified ATA (mATA) and modified EU (mEU) guidelines incorporated the size threshold suggested by the ACR guideline. *ACR* American College of Radiology^3^, *Kwak* Kwak et al.’s study^8^, *ATA* American Thyroid Association^7^, *EU* European Thyroid Association^11^.

**Figure 3:**
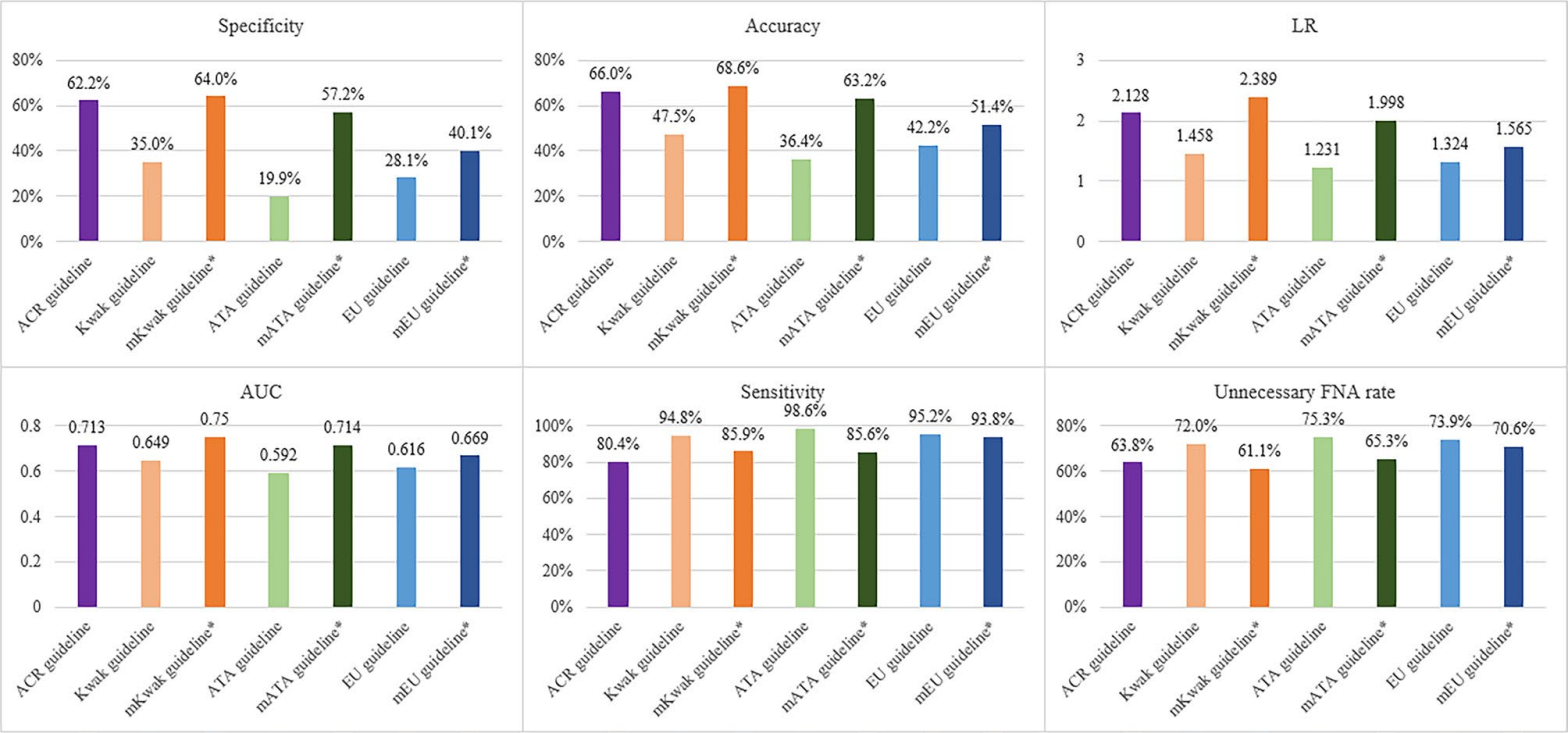
Diagnostic performances of the four guidelines and their modified guidelines. The modified Kwak (mKwak), modified ATA (mATA) and modified EU (mEU) guidelines incorporated the size threshold suggested by the ACR guideline. *ACR* American College of Radiology^3^, *Kwak* Kwak et al.’s study^8^, *ATA* American Thyroid Association^7^, *EU* European Thyroid Association^11^.

Among the original guidelines we evaluated, the ACR TIRADS had highest specificity, accuracy, LR and AUC (62.2%, 66%, 2.128 and 0.713, respectively) (P < 0.001 for all, Tables 3 and 4, Figs. 2 and 3) followed by Kwak guideline (35%, 47.5%, 1.458 and 0.649, respectively), EU guideline (28.1%, 42.2%, 1.324 and 0.616, respectively) and ATA guideline (19.9%, 36.4%, 1.231 and 0.592, respectively). Sensitivity was the highest with the ATA guideline (98.6%) and the lowest with the ACR guideline (80.4%, P = 0.011 comparing ATA and Kwak, P = 0.001 comparing the ATA and EU guidelines, P < 0.001 for the other guidelines).

**Table 3:** Diagnostic Performances of the Four Guidelines and their Modified Guidelines.

	**Sensitivity**	**Specificity**	**Accuracy**	**PPV**	**NPV**	**LR**	**AUC**
ACR guideline	80.4% (75.9–85%), [234/291]	62.2% (59.3–65.1%), [680/1093]	66% (63.5–68.5%), [914/1384]	36.2% (32.5–39.9%), [234/647]	92.3% (90.3–94.2%), [680/737]	2.128 (1.935–2.34)	0.713 (0.686–0.74)
Kwak guideline	94.8% (92.3–97.4%), [276/291]	35% (32.1–37.8%), [382/1093]	47.5% (44.9–50.2%), [658/1384]	28% (25.2–30.8%), [276/987]	96.2% (94.3–98.1%) [382/397]	1.458 (1.385–1.534)	0.649 (0.63–0.668)
mKwak guideline*	85.9% (81.9–90%), [250/291]	64% (61.2–66.9%), [700/1093]	68.6% (66.2–71.1%), [950/1384]	38.9% (35.1–42.6%), [250/643]	94.5% (92.8–96.1%), [700/741]	2.389 (2.18–2.619)	0.75 (0.725–0.774)
ATA guideline	98.6% (97.3–100%), [287/291]	19.9% (17.5–22.2%), [217/1093]	36.4% (33.9–39%), [504/1384]	24.7% (22.2–27.2%), [287/1163]	98.2% (96.4–100%), [217/221]	1.231 (1.191–1.271)	0.592 (0.579–0.606)
mATA guideline*	85.6% (81.5–89.6%), [249/291]	57.2% (54.2–60.1%), [625/1093]	63.2% (60.6–65.7%), [874/1384]	34.7% (31.2–38.2%), [249/717]	93.7% (91.9–95.5%), [625/667]	1.998 (1.839–2.172)	0.714 (0.689–0.739)
EU guideline	95.2% (92.7–97.6%), [277/291]	28.1% (25.4–30.8%), [307/1093]	42.2% (39.6–44.8%), [584/1384]	26.1% (23.4–28.7%), [277/1063]	95.6 (93.4–97.9%), [307/321]	1.324 (1.265–1.385)	0.616 (0.598–0.635)
mEU guideline*	93.8% (91–96.6%), [273/291]	40.1% (37.2–43%), [438/1093]	51.4% (48.7–54%), [711/1384]	29.4% (26.5–32.4%), [273/928]	96.1% (94.3–97.8%), [438/456]	1.565 (1.479–1.657)	0.669 (0.649–0.69)

Number in parentheses are 95% confidence intervals. Numbers in brackets are raw data.

*NPV* negative predictive value, *PPV* positive predictive value, *LR* likelihood ratio, *AUC* area under the receiver operating characteristic curve, *ACR* American College of Radiology^3^, *Kwak* Kwak et al.’s study^8^, *ATA* American Thyroid Association^7^, *EU* European Thyroid Association^11^.

*The modified Kwak (mKwak), modified ATA (mATA) and modified EU (mEU) guidelines incorporated the size threshold suggested by the ACR guideline.

**Table 4:** Comparison of Diagnostic Performances of the Four Guidelines and their Modified Guidelines.

	**P value**						
	**Sensitivity**	**Specificity**	**Accuracy**	**PPV**	**NPV**	**LR**	**AUC**
ACR vs Kwak	< .001	< .001	< .001	< .001	< .001	< .001	< .001
ACR vs mKwak*	< .001	0.014	< .001	< .001	< .001	< .001	< .001
ACR vs ATA	< .001	< .001	< .001	< .001	< .001	< .001	< .001
ACR vs mATA*	0.008	< .001	0.009	0.117	< .001	0.115	0.958
ACR vs EU	< .001	< .001	< .001	< .001	0.002	< .001	< .001
ACR vs mEU*	< .001	< .001	< .001	< .001	< .001	< .001	< .001
Kwak vs mKwak	< .001	< .001	< .001	< .001	0.028	< .001	< .001
Kwak vs ATA	0.011	< .001	< .001	< .001	0.132	< .001	< .001
Kwak vs mATA	< .001	< .001	< .001	< .001	0.003	< .001	< .001
Kwak vs EU	0.853	< .001	< .001	0.006	0.693	0.006	< .001
Kwak vs mEU	0.59	0.008	0.015	0.055	0.894	0.055	< .001
mKwak vs ATA	< .001	< .001	< .001	< .001	< .001	< .001	< .001
mKwak vs mATA	0.808	< .001	< .001	< .001	0.181	< .001	< .001
mKwak vs EU	< .001	< .001	< .001	< .001	0.211	< .001	< .001
mKWak vs mEU	< .001	< .001	< .001	< .001	0.027	< .001	< .001
ATA vs mATA	< .001	< .001	< .001	< .001	< .001	< .001	< .001
ATA vs EU	0.001	< .001	< .001	< .001	0.011	< .001	0.001
ATA vs mEU	< .001	< .001	< .001	< .001	0.021	< .001	< .001
mATA vs EU	< .001	< .001	< .001	< .001	0.088	< .001	< .001
mATA vs mEU	< .001	< .001	< .001	< .001	0.02	< .001	0.001
EU vs mEU	0.044	< .001	< .001	< .001	0.451	< .001	< .001

*NPV* negative predictive value, *PPV* positive predictive value, *LR* likelihood ratio, *AUC* area under the receiver operating characteristic curve, *ACR* American College of Radiology^3^, *Kwak* Kwak et al.’s study^8^, *ATA* American Thyroid Association^7^, *EU* European Thyroid Association^11^.

*The modified Kwak (mKwak), modified ATA (mATA) and modified EU (mEU) guidelines incorporated the size threshold suggested by the ACR guideline.

When the size threshold of ACR TIRADS was applied to the original TIRADS, the diagnostic ability increased in terms of specificity, accuracy, LR and AUC for all guidelines (Tables 3 and 4, Figs. 2 and 3). The modified Kwak (mKwak) guideline had a specificity of 64%, accuracy of 68.6%, LR of 2.389 and AUC of 0.75 while the Kwak guideline had a specificity of 35%, accuracy of 47.5%, LR of 1.458 and AUC of 0.649 (P < 0.001 for all). The modified ATA (mATA) guideline had a specificity of 57.2%, accuracy of 63.2%, LR of 1.998 and AUC of 0.714, while the original ATA guideline had a specificity of 19.9%, accuracy of 36.4%, LR of 1.231 and AUC of 0.592 (P < 0.001 for all). The modified EU (mEU) guideline had a specificity of 40.1%, accuracy of 51.4%, LR of 1.565 and AUC of 0.669, while the EU guideline had a specificity of 28.1%, accuracy of 42.2%, LR of 1.324 and AUC of 0.616 (P < 0.001 for all). However, the sensitivities of the modified guidelines were lower than their original versions. The sensitivity of the original guidelines was 94.8%, 98.6%, 95.2% for the Kwak, ATA and EU guidelines, respectively, while the modified versions showed a sensitivity of 85.9%, 85.6% and 93.8% for the mKwak, mATA and mEU guidelines, respectively. Among all the original and modified guidelines, the mKwak guideline had the highest specificity, accuracy, LR and AUC (64%, 68.6%, 2.389 and 0.75, respectively) (P = 0.014 comparing the specificity of with ACR and P < 0.001 for the others).

The unnecessary FNA rate was the lowest with the mKwak guideline (61.1%, 393/643) followed by the ACR (63.8%, 413/647), mATA (65.3%, 468/717), mEU (70.6%, 655/928), Kwak (72%, 711/987), EU (73.9%, 786/1,063) and ATA guidelines (75.3%, 876/1,163) (Table 5, Fig. 3). In all modified guidelines, the unnecessary FNA rate decreased comparing to the original guidelines when the size threshold of the ACR TIRADS was applied.

**Table 5:** Unnecessary Fine-needle Aspiration Rates.

	**Unnecessary FNA rate (%)**	**No. of FNA nodules**	**No. of test-negative nodules among FNA nodules**
ACR guideline	63.8	647	413
Kwak guideline	72	987	711
mKwak guideline*	61.1	643	393
ATA guideline	75.3	1,163	876
mATA guideline*	65.3	717	468
EU guideline	73.9	1,063	786
mEU guideline*	70.6	928	655

*FNA* Fine-Needle Aspiration, *ACR* American College of Radiology^3^, *Kwak* Kwak et al.’s study^8^, *ATA* American Thyroid Association^7^, *EU* European Thyroid Association^11^.

*The modified Kwak (mKwak), modified ATA (mATA) and modified EU (mEU) guidelines incorporated the size threshold suggested by the ACR guideline.

## Discussion

Currently, many guidelines composed of various TIRADS and size thresholds exist for further work-up such as FNA or follow-up US^3,4,7,11^. However, there has been no proven universal guideline proposed to reduce unnecessary FNAs and to find as many thyroid cancers as possible. It has also been difficult to compare the risk stratification systems themselves as each uses a different size threshold to recommend FNA although many studies have compared the diagnostic performances and unnecessary FNA rates of these guidelines^12,20–25^. To overcome this problem, we applied the size threshold of the ACR guideline to the Kwak, ATA and EU guidelines by matching the recommended malignancy rates. After applying the ACR TIRADS size threshold in the modified guidelines, diagnostic ability increased in terms of specificity, accuracy, LR and AUC compared with the original guidelines and the unnecessary FNA rates were also lower. The mKwak guideline which incorporated the ACR size threshold showed the best diagnostic results among the original and modified guidelines in terms of specificity, accuracy, LR and AUC.

Recently, many researchers demonstrated that the ACR TIRADS had superior diagnostic performance compared to other guidelines and reduced larger number of unnecessary FNAs (compared with guidelines from ATA, EU, American Association of Clinical Endocrinologists/American College of Endocrinology/Associazione Medici Endocrinologi, National Comprehensive Cancer Network, French Society of Endocrinology, Society of Radiology in Ultrasound and Korean Thyroid Association/Korean Society of Thyroid)^12,21–23,25^. Considering that the ACR incorporates a larger size threshold for FNA despite using similar recommended malignancy risks, the better diagnostic ability of the ACR guidelines can be explained by the size criteria for FNA and not the complicated US risk stratification system itself^26^. In this study, the ACR guideline showed better diagnostic accuracy than the original Kwak guideline which uses a 10 mm size threshold to recommend US-guided FNA (US-FNA) regardless of the number of suspicious US features. However, the mKwak guideline showed higher diagnostic accuracy than the original ACR guideline after the size threshold of the ACR guideline was applied. When US risk stratification systems are compared between the ACR and Kwak guidelines, the Kwak guideline is more straightforward and practical to use than the ACR guideline which uses a different point system for individual US features as they are assigned different weights^3,8^. Therefore, a combination of the easier US risk stratification system of the Kwak guideline and the size threshold of the ACR guideline can help clinicians in daily practice.

Increasing the size threshold of US-FNA resulted in decreasing the unnecessary FNA rate in all the guidelines we evaluated, which was the trade-off for lower sensitivity. In our study, the unnecessary FNA rate decreased more than sensitivity did for both the Kwak and EU guidelines. Size modification reduced the unnecessary FNA rate of the Kwak and EU guidelines by 10.9% and 3.3%, respectively while reducing sensitivity by 8.9% and 1.4%, respectively. When the ATA and mATA guidelines were compared, sensitivity decreased by 13% and the unnecessary FNA rate decreased by 10% with the mATA guidelines. As the only difference between the modified and original guidelines was size criteria, we can assume that the size threshold proposed by the ACR guideline increased diagnostic accuracy and reduced the unnecessary FNA rates. In one recent study, diagnostic performance and the unnecessary biopsy rate were evaluated with simulations using various nodule size cutoffs applied to the ATA and Korean Thyroid Association/Korean Society of Thyroid Radiology guidelines (KTA/KSThR)^22^. Among the various simulations, the 15 mm cutoff for intermediate suspicion, 25 mm cutoff for low suspicion and eliminating FNA for nodules of very low suspicion in the ATA guideline showed the highest specificity, accuracy and the lowest unnecessary biopsy rate^22^. These results suggest that the high specificity and low unnecessary FNA rate of the ACR guideline was due to the larger size cutoff which is in line with our study results^22^.

There are several limitations to this study. First, 1,244 of the 1,384 thyroid nodules (89.9%) were diagnosed based on cytologic findings alone, which could have resulted in some missed malignancies. We only included the nodules with definitive diagnostic cytopathologic findings (benign or malignant) at US-FNA, core needle biopsy, or surgery. Also, 5.2% (21/396) of the follicular carcinomas were diagnosed after surgery. Thus, a selection bias exists. Second, an experienced radiologist retrospectively re-assigned categories to thyroid nodules according to different risk stratification systems using US features prospectively recorded by 14 radiologists who were familiar with point-scale risk stratification. When US descriptors were recorded in this study, they could not be defined with the exact same definitions used in the other original guidelines, an issue which was not considered during data analysis, and this might have led to differences in the final assessments made in real-time examinations. Reassigning categories previously assigned according to the point-scale system to categories based on the pattern-recognition system might have also affected the results of this study. Third, the 14 radiologists performing the prospective imaging acquisition and analysis had variable levels of experience. Although interobserver variability and consistency are important considerations for choosing appropriate guidelines^27,28^, our study is reflective of actual clinical practice. Forth, the relatively high malignancy rate of thyroid nodules in our study is probably because we only included thyroid nodules which underwent FNA, which would naturally lead to a higher number of malignant nodules. Also, our institution is a tertiary referral center and that itself is a reason for the high malignancy rate of the study population.

In conclusion, application of the larger US-FNA size threshold of the ACR guideline resulted in increased diagnostic accuracy and decreased unnecessary FNA rates at the expense of decreased sensitivity. The mKwak guideline which is practical and easy to use showed superior diagnostic accuracy than the other guidelines, both original and modified. Further longitudinal multicenter studies with larger data are needed in the future to choose an accurate and effective risk stratification system for daily practice.

## Methods

The institutional review board (IRB) of the Yonsei University College of Medicine approved this retrospective study and the requirement for informed consent for review of images and medical records was waived. And all methods were performed in accordance with the Declaration of Helsinki.

### Study cohort

This study was performed from December 2015 to November 2016, during which 2,179 patients underwent US-FNA to diagnose thyroid nodules at our institution, a tertiary referral center. Among them, a total of 1704 thyroid nodules in 1602 patients were 10 mm or larger on US. 320 nodules were excluded because of a lack of definitive cytopathologic results after being initially diagnosed as nondiagnostic (n = 176), atypia or follicular lesion of undetermined significance (n = 110), follicular neoplasm or suspicion of follicular neoplasm (n = 27), or suspicion of malignancy (n = 7). Nodules were included if they had definitive diagnostic cytopathologic findings (benign or malignant) at US-FNA, core needle biopsy, or surgery. Finally, 1,384 thyroid nodules in 1,301 patients were included (Fig. 1).

Mean age of the 1,301 patients was 50.2 ± 13.6 years old (range 18–90 years). Mean size of the 1,384 thyroid nodules was 23.2 ± 12.6 mm (range 10-100 mm). Of the total patients, 1,062 (81.6%) were women and 239 (18.4%) were men. Of the total patients, 77 had two nodules and three had three nodules.

### US examinations

Thyroid US was performed with a 5–12 MHz linear array transducer (iU22; Philips Medical Systems). US examinations were performed by one of 14 board-certified radiologists (5 faculties and 9 fellows) with 1–20 years of experience in thyroid imaging. US-FNAs were subsequently performed by the same radiologist who performed the thyroid US examination.

US features of thyroid nodules which underwent US-FNA were prospectively described and recorded in our institutional database at the time of US-FNA by the radiologist who performed the US and US-FNA according to composition, echogenicity, margin, calcifications, and shape. The composition was classified as solid, predominantly solid, predominantly cyst, spongiform nodule and cyst, the echogenicity was classified as hyperechogenicity, isoechogenicity, hypoechogenicity and marked hypoechogenicity, the margin was classified as well-defined, microlobulated and irregular margin, the calcification was classified as negative, egg-shell calcification, macrocalcification, microcalcification and mixed calcification. And the shape was classified as parallel and non-parallel. At our institution, US findings of solid composition, hypoechogenicity or marked hypoechogenicity, microlobulated or irregular margins, microcalcifications, and nonparallel shape were considered to be suspicious features for malignancy^29^.

### Data and statistical analysis

Cytopathology results from FNA and surgery were considered as the standard reference. One radiologist (J.Y.K) with 17 years of experience in thyroid imaging, blind to the patients’ clinical data and pathological results, retrospectively re-assigned the TIRADS categories of each thyroid nodule using our institutional database which was made up of data collected by the radiologists who performed the US-FNAs. Ninety thyroid nodules (6.5%, 90/1,384) unspecified according to the ATA guideline including isoechoic or hyperechoic nodules with suspicious US features^7^ were regarded as intermediate suspicion as the calculated malignancy rates of these nodules were within the range of 10–20%^30^.

Indications for FNA were based on US features and lesion size according to the various guidelines we used in this study^3,7,11^. A size threshold of 10 mm was used to indicate US-FNA in all thyroid nodules with suspicious US features in the Kwak TIRADS because the Kwak TIRADS recommends US-FNA when thyroid nodules more than 10 mm in size have suspicious US features rather than applying different size thresholds according to the final assessment category^8,29^. We applied the size criteria of the ACR TIRADS to the Kwak, ATA and EU guidelines according to similar recommended malignancy risk of each category^3,7,8,11^, and defined the new guidelines as the mKwak, mATA and mEU guidelines, respectively (Supplementary Table S1 online). The ACR TIRADS recommends no FNA for not suspicious thyroid nodules with recommended risk of malignancy of 2%^3^. The same strategy was applied for very low suspicion category of ATA guideline with recommended risk of malignancy of less than 3%^7^. For mildly suspicious thyroid nodules with a recommended malignancy risk of 5% in the ACR TIRADS, FNA was recommended when the nodule was 25 mm or larger^3^. The same size threshold was applied for nodules of low risk according to the EU guideline rather than the present size threshold of 20 mm because the recommended risks of malignancy was 2–4%^11^. The recommended malignancy risk was 5–20% for moderately suspicious nodules in the ACR TIRADS and FNA was recommended when the nodule was 15 mm or larger^3^. A size threshold of 15 mm was applied instead of 10 mm for nodules of intermediate suspicion according to the ATA guideline with a recommended malignancy risk of 10–20%^7^. We also used a size threshold proposed by the ACR TIRADS to the Kwak guideline^3,8^: 25 mm size threshold for category 4a, 15 mm for category 4b and 10 mm for category 4c and 5. As the spongiform nodule and isolated macrocalcifications have no suspicious US feature according to Kwak TIRADS, they are considered as category 3^8^.

Thyroid nodules were classified as nodules for which US-FNA was indicated and those for which it was not, according to the FNA criteria provided by each guideline^3,7,8,11^.

To compare the demographics between benign and malignant nodules, the independent two sample t-test was used to compare continuous data including patient age and the Chi-square test was used to compare categorical data including patient sex. Since some patients had more than one nodule, the generalized estimated equation (GEE) was used to compare both continuous and categorical data between benign and malignant nodules. Malignancy rates according to the final assessment by each system were calculated and compared with GEE. We also evaluated diagnostic performances including sensitivity, specificity, accuracy, negative predictive value (NPV), positive predictive value (PPV), likelihood ratio (LR) and area under the receiver operating characteristic curve (AUC) along with 95% confidence intervals (CI). The sensitivity, specificity, accuracy, NPV, PPV and LR were compared with GEE. The Delong method was used to compare AUC. The unnecessary biopsy rate for the diagnosis of thyroid cancer was defined as the number of benign nodules among the biopsy-required nodules. Statistical analysis was performed with SAS software (version 9.4, SAS Inc.). A two-sided P < 0.05 was considered to indicate statistical significance.

## Supplementary information

Supplementary information
