# Supplementary material for: Diagnostic performances and unnecessary US-FNA rates of various TIRADS after application of equal size thresholds
Source: Sci Rep. 2020 Jun 30;10:10632. doi: 10.1038/s41598-020-67543-z (PMC7326914; doi:10.1038/s41598-020-67543-z)
Supplement: Supplementary file 1 — Supplementary information [file 41598_2020_67543_MOESM1_ESM.docx]

**Diagnostic Performances and Unnecessary US-FNA Rates of Various TIRADS after Application of Equal Size Thresholds**

Sun Huh, M.D.^1^, Hye Sun Lee, Ph.D,^2^ Jiyoung Yoon, M.D. ^1^, Eun-Kyung Kim, M.D., Ph.D^1^, Hee Jung Moon, M.D., Ph.D^1^_,_ Jung Hyun Yoon, M.D., Ph.D^1^, Vivian Youngjean Park, M.D., Ph.D^1^, *Jin Young Kwak, M.D., Ph.D^1^

^1^Department of Radiology and Research Institute of Radiological Science, Severance Hospital, Yonsei University College of Medicine, ^2^Biostatistics Collaboration Unit, Yonsei University College of Medicine

**Corresponding author: Jin Young Kwak, M.D., Ph.D**

Department of Radiology, Severance Hospital, Yonsei University, College of Medicine
50-1 Yonsei-ro Seodaemun-gu, Seoul 03722, Korea
Tel.: 82-2- 2228-7400

Fax: 82-2-2227-8337
E-mail: [docjin@yuhs.ac](mailto:docjin@yuhs.ac)

**Table S1. Size threshold of the four guidelines and their modified guidelines for fine-needle aspiration**

|  | **No FNA** | **FNA ≥ 25mm** | **FNA ≥ 15mm** | **FNA ≥ 10mm** |
| --- | --- | --- | --- | --- |
| ACR guideline^*^ | 2 – not suspicious  2 points | 3 – mildly suspicious  3 points | 4 - moderately suspicious  4 to 6 points | 5 – highly suspicious  7 points or more |
| mKwak guideline^‡^ | 3 – no suspicious US feature | 4a – one suspicious US feature | 4b – two suspicious US feature | 4c – three or four suspicious US feature  5 – five suspicious US features |
| mATA guideline^‡^ | Benign  2 – very low suspicion |  | 3 – low suspicion  4 – intermediate suspicion | 5 – high suspicion |
| mEU guideline^‡^ | Benign | 3 – low risk | 4 – intermediate risk | 5 – high risk |
|  | **No FNA** | **FNA ≥ 20mm** | **FNA ≥ 15mm** | **FNA ≥ 10mm** |
| Kwak guideline^†^ | 3 – no suspicious US feature |  |  | 4a – one suspicious US feature  4b – two suspicious US feature  4c – three or four suspicious US feature  5 – five suspicious US features |
| ATA guideline | **Benign**  Pure cystic nodules | **2 – very low suspicion**  Spongiform or partially cystic nodules without any of the sonographic features described in low, intermediate or high suspicion patterns | **3 – low suspicion**  Isoechoic or hyperechoic solid nodule, or partially cystic nodule with eccentric solid areas, without microcalcification, irregular margin or ETE, or taller than wide shape | **4 – intermediate suspicion**  Hypoechoic solid nodule with smooth margins without microcalcifications, ETE, or taller than wide shape  **5 – high suspicion**  Solid hypoechoic nodule or solid hypoechoic component of partially cystic nodule with one or more of the following features: irregular margins, microcalcifications, taller than wide shape, rim calcifications with small extrusive soft tissue component, evidence of ETE |
| EU guideline | **Benign**  Pure cyst or entirely spongiform | **3 – low risk**  Ovoid, smooth isoechoic/hyperechoic without features of high suspicion | **4 – intermediate risk**  Ovoid, smooth, mildly hypoechoic without features of high suspicion | **5 – high risk**  At least one of high suspicion; irregular shape, irregular margin, microcalcifications, marked hypoechogenicity |

^*^Points are given for all US features in a nodule, with more suspicious features being awarded additional points in the ACR guideline and the total sum determines the nodule’s TIRADS level. When assessing a nodule, the reader selects a feature from each category (composition, echogenicity, shape, margin and echogenic foci). For composition, mixed cystic and solid is given one point and solid or almost completely solid two. For echogenicity, hyperechogenicity or isoechogenicity is given one point, hypoechogenicity two and very hypoechogenicity three. For shape, taller-than-wide shape is given three points. For margin, lobulated or irregular margins are given one point and extra-thyroidal extension three. For echogenic foci, the reader chooses all features that apply, with one point given for macrocalcifications, two for peripheral (rim) calcifications and three for punctate echogenic foci.

^†^Suspicious features include solid composition, hypoechogenicity or marked hypoechogenicity, irregular or microlobulated margin, microcalcifications or mixed calcifications and taller-than-wide shape

^‡^The modified Kwak (mKwak), modified ATA (mATA) and modified EU (mEU) guidelines incorporated the size threshold suggested by the ACR guideline.

ACR = American College of Radiology [3], Kwak = Kwak et al.’s study [8], ATA = American Thyroid Association [7], EU = European Thyroid Association [11], ETE = extrathyroidal extension
